# Spectrum of pathogen- and model-specific histopathologies in mouse models of acute pneumonia

**DOI:** 10.1371/journal.pone.0188251

**Published:** 2017-11-20

**Authors:** Kristina Dietert, Birgitt Gutbier, Sandra M. Wienhold, Katrin Reppe, Xiaohui Jiang, Ling Yao, Catherine Chaput, Jan Naujoks, Markus Brack, Alexandra Kupke, Christin Peteranderl, Stephan Becker, Carolin von Lachner, Nelli Baal, Hortense Slevogt, Andreas C. Hocke, Martin Witzenrath, Bastian Opitz, Susanne Herold, Holger Hackstein, Leif E. Sander, Norbert Suttorp, Achim D. Gruber

**Affiliations:** 1 Department of Veterinary Pathology, Freie Universität Berlin, Berlin, Germany; 2 Department of Infectious Diseases and Pulmonary Medicine, Charité-Universitätsmedizin Berlin, Berlin, Germany; 3 Department of Internal Medicine II, Section for Infectious Diseases, Universities Giessen & Marburg Lung Center (UGMLC), Member of the German Center for Lung Research (DZL) Giessen, Germany; 4 Institute of Virology, Philipps University of Marburg, German Center for Infection Research (DZIF), TTU Emerging Infections, Marburg, Germany; 5 Septomics Research Center, Jena University Hospital, Jena, Germany; 6 Institute for Clinical Immunology and Transfusion Medicine, Universities of Giessen and Marburg Lung Center (UGMLC), Member of the German Center for Lung Research (DZL), University Hospital Giessen und Marburg, Justus-Liebig-University Giessen, Giessen, Germany; Louisiana State University, UNITED STATES

## Abstract

Pneumonia may be caused by a wide range of pathogens and is considered the most common infectious cause of death in humans. Murine acute lung infection models mirror human pathologies in many aspects and contribute to our understanding of the disease and the development of novel treatment strategies. Despite progress in other fields of tissue imaging, histopathology remains the most conclusive and practical read out tool for the descriptive and semiquantitative evaluation of mouse pneumonia and therapeutic interventions. Here, we systematically describe and compare the distinctive histopathological features of established models of acute pneumonia in mice induced by *Streptococcus (S*.*) pneumoniae*, *Staphylococcus aureus*, *Klebsiella pneumoniae*, *Acinetobacter baumannii*, *Legionella pneumophila*, *Escherichia coli*, Middle East respiratory syndrome (MERS) coronavirus, influenza A virus (IAV) and superinfection of IAV-incuced pneumonia with *S*. *pneumoniae*. Systematic comparisons of the models revealed striking differences in the distribution of lesions, the characteristics of pneumonia induced, principal inflammatory cell types, lesions in adjacent tissues, and the detectability of the pathogens in histological sections. We therefore identified core criteria for each model suitable for practical semiquantitative scoring systems that take into account the pathogen- and model-specific patterns of pneumonia. Other critical factors that affect experimental pathologies are discussed, including infectious dose, time kinetics, and the genetic background of the mouse strain. The substantial differences between the model-specific pathologies underscore the necessity of pathogen- and model-adapted criteria for the comparative quantification of experimental outcomes. These criteria also allow for the standardized validation and comparison of treatment strategies in preclinical models.

## Introduction

As one of the most frequent infectious diseases, pneumonia causes a tremendous socioeconomic burden in industrialized countries [1] and is the leading infectious cause of death in children worldwide [2]. Numerous classes of pathogens can cause acute pneumonia [3] and the risk of pneumonia is greatly enhanced under conditions of impaired pulmonary host defense, including preceding viral infections [4], mechanical ventilation [5] and sepsis [6]. The leading causative pathogen of community acquired pneumonia (CAP) is the Gram-positive bacterium *Streptococcus (S*.*) pneumoniae* [7, 8] which accounts for the majority of bacterial upper and lower respiratory tract infections and is responsible for millions of deaths annually [9, 10]. As another cause of CAP influenza A virus (IAV) infection leads to rapid progression of lung failure with limited treatment options and frequent fatal outcome [3, 11, 12]. Moreover, IAV infections are commonly complicated by bacterial superinfection, mostly caused by *S*. *pneumoniae*, resulting in severe progressive pneumonia associated with increased mortality [13]. In contrast, the Gram-negative and facultatively intracellular bacterium *Legionella (L*.*) pneumophila* is the causative agent of the severe CAP Legionnaires’ disease, and the second most commonly detected pathogen in pneumonia in patients admitted to intensive care units (ICU) in industrialized countries [14, 15]. However, in addition to CAP, ventilator-associated pneumonia (VAP) is also a major cause of hospital morbidity and mortality in ICUs [16] and the spectrum of pathogens is shifted in these forms of pneumonia. Here *Staphylococcus (S*.*) aureus*, *Klebsiella (K*.*) pneumoniae*, *Acinetobacter (A*.*) baumannii*, and *Escherichia (E*.*) coli* have been isolated with varying prevalences [17–19]. More specifically, the Gram-negative *K*. *pneumoniae* is a significant opportunistic pathogen causing severe life-threatening hospital-acquired respiratory tract infections [20–22] while *S*. *aureus*, a Gram-positive bacterium, is one of the most prevalent pathogens of community- and hospital-acquired lower respiratory tract infections in humans and accounts for a significant health and economic burden [23–25]. *A*. *baumannii* and *E*. *coli* are ubiquitous Gram-negative bacteria which have recently emerged as major causes of community-associated, nosocomial [26, 27] and ventilator-associated pneumonia [19, 28] as well as septicemia induced acute lung injury (ALI) [29, 30].

In addition, more recently discovered pulmonary pathogens indicate that novel emerging diseases may add to the list of highly relevant pneumonias that may also be of interest to be studied in animal models. For example, the Middle East respiratory syndrome coronavirus (MERS-CoV) which is transmitted by dromedary camels as vectors [31] has emerged as the cause of severe human respiratory disease worldwide [32, 33] with elderly and immunocompromised individuals particularly in Saudi Arabia being at highest risk [34–36].

The various forms of pneumonia have been successfully reproduced in specific murine models of experimentally-induced acute pneumonia [37–39]. These models have substantially contributed to our understanding of the pathogenesis of community- and hospital-acquired pneumonia as well as emerging lung infections worldwide and are indispensable for the development of novel therapeutic strategies [40–42].

Histopathology has been a powerful, reliable, and reproducible read-out tool for the evaluation of morphological changes in animal lung infection experiments for many decades [43, 44]. Qualitative diagnoses are based on a summation of microscopically observable changes in the morphology and cellular composition of the tissue and cell types involved. For a more comparative inclusion of histopathologic information in biomedical research, scoring systems have been widely applied which allow for a first semiquantitative assessment of lesions compared to controls [44, 45]. Moreover, all preclinical models used for the development of novel treatment strategies and acceptance by regulatory agencies need to be assessed histopathologically by board certified pathologists as gold standard for qualitative and semiquantitative evaluation of tissue alterations in experimental animals [46–48].

Previous studies have revealed first fundamental differences in histopathologic lesions caused by different pathogens in mouse lungs [38, 41, 42]. However, scoring schemes for acute murine pneumonia existing to date are very superficial, addressing only a few, rather unspecific parameters [45, 49, 50]. More importantly, they hardly allow for a differentiating perspective between distinct pathogens or for group comparisons, e.g., infections of wild type versus genetically modified mice. Clearly, there is a strong need for more precise and pathogen- as well as model-specific parameters to allow for an accurate description and semiquantification of the inflammatory phenotype for reliable and reproducible comparisons between experimental groups within each model. Therefore, we have recently adapted more specific scoring criteria for *S*. *pneumoniae* and *S*. *aureus-*induced pneumonia [38, 42]. However, such pathogen-specific scoring criteria have not been employed for other lung pathogens in mice.

Here, we systematically describe and compare the histopathologies at their peaks of inflammation and injury of nine previously established acute lung infection models induced by *S*. *pneumoniae*, *S*. *aureus*, *K*. *pneumoniae*, *A*. *baumannii*, *L*. *pneumophila*, *E*. *coli*, MERS-CoV, IAV and superinfection with IAV and pneumococci. We provide model-specific criteria that can be used for appropriate histological quantitative comparisons, e.g., when different therapeutic interventions are evaluated within these established models. Whole mouse lung sections were used to obtain complete overviews, particularly of the distributions of lesions and inflammatory patterns. On the basis of the different and oftentimes quite pathogen- and model-specific changes we identified the most suitable evaluation criteria for each model that will allow for more accurate semiquantitative assessments of the severities and distributions of pneumonic lesions.

## Materials and methods

### Ethics statement

The lung tissues examined here were derived from experiments primarily conducted for purposes other than this study and most have been published elsewhere [38, 41, 42, 51–53], except for the *A*. *baumannii* and *E*. *coli* experiments that will be published elsewhere. All animal procedures and protocols were approved by institutional ethics committees of Charité-University of Berlin, Justus-Liebig University of Giessen, Philipps University of Marburg, University Hospital of Jena and local governmental authorities (Landesamt für Gesundheit und Soziales (LAGeSo) Berlin, Regierungspräsidium (RP) Gießen and Darmstadt, Landesamt für Verbraucherschutz (TLV) Thüringen), respectively. Permit numbers were G 0356/10, A-0050/15 (*S*. *pneumoniae*), G 0358/11 (*S*. *aureus*), G 75/2011, G 110/2014 (*K*. *pneumoniae*), A 0299/15 (*A*. *baumannii*), G 0175/12 (*L*. *pneumophila*), 02-049/12 (*E*. *coli*), 114/2012 (MERS-CoV), G 0152/12, and G 0044/11 (IAV and superinfection). All animal studies were conducted in strict accordance with the Federation of European Laboratory Animal Science Associations (FELASA) guidelines and recommendations for the care and use of laboratory animals, and all efforts were made to minimize animal discomfort and suffering.

All mice, except for MERS-CoV infected mice, were monitored at 12 hour intervals throughout the experiment to assess appearance, behavior, grooming, respiration, body weight, and rectal temperature. Humane endpoints were defined (body temperature <30°C, body weight loss = 20%, cumbersome breathing, accelerated breathing in combination with staggering, pain or paleness) but not reached by any of the mice at the indicated time points of termination of the experiments. MERS-CoV infected mice were monitored once daily and appearance, behavior, grooming, respiration and body weight were protocolled. Here, a single humane endpoint (loss of body weight of >15%) was defined but not reached by any of the mice employed due to their favourable clinical outcome at the infection dose used.

### Mice

For all experimental infection models, except of *K*. *pneumoniae*, female mice (aged 8–12 weeks and weighing 17–22 g) were randomly assigned to groups (n = 2–4) per cage whereas in the *K*. *pneumoniae* model female and male mice (aged 23–25 weeks and weighing 22–24 g) were used for model specific reasons [41]. Furthermore, for all experimental infection models, specific-pathogen-free (SPF) mice on C57BL/6 (all except for MERS-CoV) or BALB/c (as previously used for the MERS-CoV model [52, 54]) background were used and housed in individually ventilated cages under SPF conditions with a room temperature of 22 ± 2°C and a relative humidity of 55 ± 10%. A 12 hour light/ 12 hour dark cycle was maintained and the animals had unlimited access to standard pellet food and tap water. All experimental details of the infection models compared here were applied following previously published and well established protocols that partly vary in terms of infection doses, routes of infection and time point of examination due to pathogen- or model specific reasons, as given below.

For bacterial infections, except for *E*. *coli*, mice were anesthetized intraperitoneally with ketamine (80 mg/kg) (Ketavet; Pfizer, Berlin, Germany) and xylazine (25 mg/kg) (Rompun; Bayer, Leverkusen, Germany). For experimental viral infections, mice were anesthetized using inhalation of isoflurane (Forene; Abbott, Wiesbaden, Germany). For lung histology, all mice except of the MERS-CoV model were humanely euthanized by exsanguination via the caudal *Vena cava* after anesthesia by intraperitoneal injection of premixed ketamine (160 mg/kg) and xylazine (75 mg/kg). MERS-CoV infected mice were humanely euthanized by cervical dislocation after isoflurane anesthesia.

### Bacterial infections

*S*. *pneumoniae* (serotype 3 strain, NCTC 7978), *S*. *aureus* Newman (NCTC 10833), *K*. *pneumoniae* (serotype 2, ATCC 43816), *A*. *baumannii* (RUH 2037), *L*. *pneumophila* (serogroup 1 strain, JR 32) were cultured as described [37, 38, 40, 55, 56] and resuspended in sterile PBS. Mice were anesthetized intraperitoneally (i.p.) with ketamine (80 mg/kg) and xylazine (25 mg/kg) and transnasally inoculated with 5 x 10^6^ CFU *S*. *pneumoniae* (n = 14 mice), 5 x 10^7^ CFU *S*. *aureus* (n = 4), 5 x 10^8^ CFU *A*. *baumannii* (n = 8), in 20 μl PBS. Mice transnasally infected with *L*. *pneumophila* (n = 8) received 1 × 10^6^ CFU in 40 μl PBS. Mice infected with *K*. *pneumoniae* (n = 16) received 3.5 x 10^5^ CFU intratracheally in 50 μl NaCl (0.9%) via Microsprayer® Aerosolizer (Model IA-1b, Penn-Century, Inc., Wyndmoor, PA) using intubation-mediated intratracheal instillation through intact airways [57] which has previously been optimized for this model [41, 57–59].

*E*. *coli* (ATCC 25922) from -80°C glycerol stock was added to LB broth (Carl Roth, Karlsruhe, Germany) and incubated for 12 hours at 200 rpm and 37°C with 5% CO_2_. Optical density of 0.03 was adjusted in LB broth followed by incubation until midlogarithmic phase for 1.5 hours at 200 rpm and 37°C. After centrifugation, the pellet was resuspended in sterile 0.9% NaCl at 8 x 10^5^ CFU *E*. *coli* / 200 μl and administered intraperitoneally (n = 10).

### Viral infections and superinfection

For initial transduction of human DPP4 for subsequent infection of BALB/c mice with MERS-CoV (hCoV EMC) viruses were cultured and prepared as described [52, 54, 60]. Mice were transduced transnasally with 20 μl of an adenovirus vector encoding human DPP4 and mCherry with a final titer of 2.5 x 10^8^ PFU per inoculum (AdV-hDPP4; ViraQuest Inc.), resulting in hDPP4 expression in the epithelial compartment of the lung [60] and transnasally infected with a final titer of 7 x 10^4^ TCID_50_ of MERS-CoV in 20 μl DMEM (n = 17) under isoflurane anesthesia (Forene; Abbott, Wiesbaden, Germany).

Influenza A/PR/8/34 virus (H1N1; PR8) was grown as described [42] and mice were transnasally infected with 100 PFU PR8 in 50 μl PBS (n = 4) under isoflurane anesthesia.

For superinfection experiments, the IAV infection procedure was applied as described above with 40 PFU PR8 in 50 μl PBS. 8 days after viral infection, *S*. *pneumoniae* was cultured as described [37] and resuspended in sterile PBS. Mice were anesthetized intraperitoneally and transnasally inoculated with 1 x 10^3^ CFU *S*. *pneumoniae* in 20 μl PBS (n = 4).

### Histopathology

Mice were humanely euthanized at model-specific time points as indicated (Table 1). Between 2 and 6 repetitions of the entire experimental procedures were performed in each model with similar group sizes in each repetition. Lungs were carefully removed after ligation of the trachea to prevent alveolar collapse, immersion-fixed in formalin pH 7.0 for 24 to 48 hours (MERS-CoV for 7 days), embedded in paraffin, cut in 2 μm sections and stained with hematoxylin and eosin (HE) after dewaxing in xylene and rehydration in decreasing ethanol concentrations. Bacteria were visualized using the Giemsa and Gram (modified by Brown and Brenn) stains. For the display of whole lung overviews, HE stained slides of entire lung sections were automatically digitized using the Aperio CS2 slide scanner (Leica Biosystems Imaging Inc., CA, USA) and image files were generated using the Image Scope Software (Leica Biosystems Imaging Inc.).

**Table 1:** Comparison of relevant mouse models of acute pneumonia.

Parameter	*Streptococcus pneumoniae*	*Staphylococcus aureus*	*Klebsiella pneumoniae*	*Acinetobacter baumannii*	*Legionella pneumophila*	*Escherichia coli*	Middle East respiratory syndrome coronavirus	Influenza A virus (IAV)	IAV + *Streptococcus pneumoniae*
**Infectious dose**	5 x 10^6^ CFU	5 x 10^7^ CFU	3.5 x 10^5^ CFU	5 x 10^8^ CFU	1 x 10^6^ CFU	5 x 10^8^CFU	7 x 10^4^ TCID _50_	100 PFU	H1N1: 40 PFU, ST3: 1 x 10^3^CFU
**Route of infection**	Transnasal	Transnasal	Intratracheal	Transnasal	Transnasal	Intraperitoneal	Transnasal	Transnasal	Transnasal
**Examination** **post infection at**	48h	48h	48h	48h	48h, 6d	12h	4d	7d	8d + 24h
**Distribution of lesions**	Multifocal	Multifocal	Multifocal	Multifocal	Multifocal	Diffuse	Multifocal	Diffuse	Diffuse
**Expansion to** **lung periphery**	Yes	No	Yes	Yes	No	Yes	Yes	Yes	Yes
**Type of pneumonia**	Broncho- pneumonia	Broncho- pneumonia	Broncho- pneumonia	Broncho- pneumonia	Interstitial pneumonia	Interstitial pneumonia	Interstitial pneumonia	Bronchointerstitial pneumonia	Broncho- pneumonia
**Main character**	Suppurative	Suppurative	Suppurative	Suppurative	Histiocytic at 48h, granulomatous at 6d	Suppurative	Necrotizing	Necrotizing	Suppurative
**Dominant inflammatory** **cell type**	Neutrophils	Neutrophils / macrophages	Neutrophils	Neutrophils / macrophages	Macrophages	Neutrophils	Macrophages / lymphocytes	Lymphocytes / macrophages	Neutrophils / lymphocytes / macrophages
**Abscess formation**	-	+	+	+	-	-	-	-	-
**Alveolar edema**	+	+	+	+	-	-	+	+	+
**Hemorrhage**	+	+	+	+	-	-	+	+	+
**Vascular thrombosis**	-/+	-	-	+	-	+	+	-	-
**Pleuritis**	+	-	+	-	-	-	-	-	+
**Steatitis**	+	-	+	-	-	-	-	-	+
**Visibility of pathogens** **by H&E stain**	+	-	+	-	-	+	-	-	-

Three evenly distributed whole-organ horizontal sections throughout the entire lungs were microscopically evaluated to assess the distribution and character of pathologic alterations, generating a modified panel of specific lung inflammation parameters adapted to each pathogen used (Table 1 and Table 2). All examinations were performed by trained veterinary experimental pathologists.

**Table 2:** Pathogen and model-specific presence of lesion patterns in mouse models of acute pneumonia.

Evaluation criteria	*S*. *pneumo-niae*	*S*. *aureus*	*K*. *pneumo-niae*	*A*. *bauman-nii*	*L*. *pneumo-phila*	*E*. *coli*	MERS-CoV	IAV	IAV + *S*. *pneumo-niae*
**Expansion to peripheral lung regions**	++	-	++	++	-	diffuse	++	++	++
**Pulmonary atelectasis**	++	++	+	++	-	-	+	+	++
**Peribronchial inflammation**	++	+	+	+	+	-	+	++	++
**Perivascular inflammation**	++	+	+	+	+	+	+	+	++
**Interstitial inflammation**	+	+	+	+	++	++	++	++	++
**Intraalveolar inflammation**	++	++	++	++	+	-	+	+	++
**Bronchial epithelial cell necrosis**	+	+	+	+	-	-	+	++	++
**Alveolar wall necrosis**	++	+	+	+	+	+	++	++	++
**Infiltration by neutrophils**	++	++	++	++	+	++	++	+	++
**Infiltration by macrophages**	+	++	+	+	++	-	++	++	+
**Infiltration by lymphocytes**	-	+	-	-	+	-	++	++	++
**Abscess formation**	-	++	++	+	-	-	-	-	-
**Granuloma formation**	-	-	-	-	++	-	-	-	-
**Alveolar edema**	++	+	++	+	-	-	+	++	++
**Perivascular edema**	++	-	++	+	+	-	-	+	+
**Perivascular lymphocytic cuffing**	-	++	-	+	++	-	++	++	+
**Hyperplasia of type II alveolar epithelial cells**	-	-	-	-	-	-	+	++	+
**Vasculitis**	++	-	+	-	+	+	++	-	+
**Fibrinoid degeneration of vascular walls**	-	-	-	-	-	-	++	-	-
**Vascular thrombosis**	+/-	-	-	++	-	++	++	-	-
**Hemorrhage (interstitial, intraalveolar)**	++	+	+	+	-	-	++	+	+
**Pleuritis**	++	-	++	-	-	-	-	-	++
**Steatitis of mediastinal adipose tissue**	++	-	++	-	-	-	-	-	++
Suggested method for visualization of pathogens*	HE, Gr	Gr	HE, Gi	Gr Gi	Gr Gi	Gr Gi	IHC	IHC	IHC Gr

-, absent; +, minor; ++, major

* HE, Hematoxylin and Eosin; Gr, Gram; Gi, Giemsa stain; IHC, immunohistochemistry

### Immunohistochemistry

For immunohistochemical detection of *S*. *pneumoniae* and IAV (H1N1), antigen retrieval was performed with microwave heating (600 W) in 10 mM citric acid (750 ml, pH 6.0) for 12 minutes (min). Lung sections were then incubated with a purified rabbit antibody polyclonal to *S*. *pneumoniae* (1:2,000, kindly provided by S. Hammerschmidt) or with a purified goat antibody polyclonal to IAV H1N1 (1:4,000, OBT155, Bio-Rad, Puchheim, Germany) at 4°C overnight. Incubation with an immuno-purified rabbit or goat antibody at the same dilution served as negative controls. Subsequently, slides were incubated with a secondary, alkaline phosphatase-conjugated goat anti-rabbit (1:500, AP-1000, Vector, Burlingame, CA) antibody for 30 min at room temperature. The alkaline chromogen triamino-tritolyl-methanechloride (Neufuchsin) was used as phosphatase substrate for color development. All slides were counterstained with hematoxylin, dehydrated through graded ethanols, cleared in xylene and coverslipped.

## Results

### S. pneumoniae

**Fig 1:**
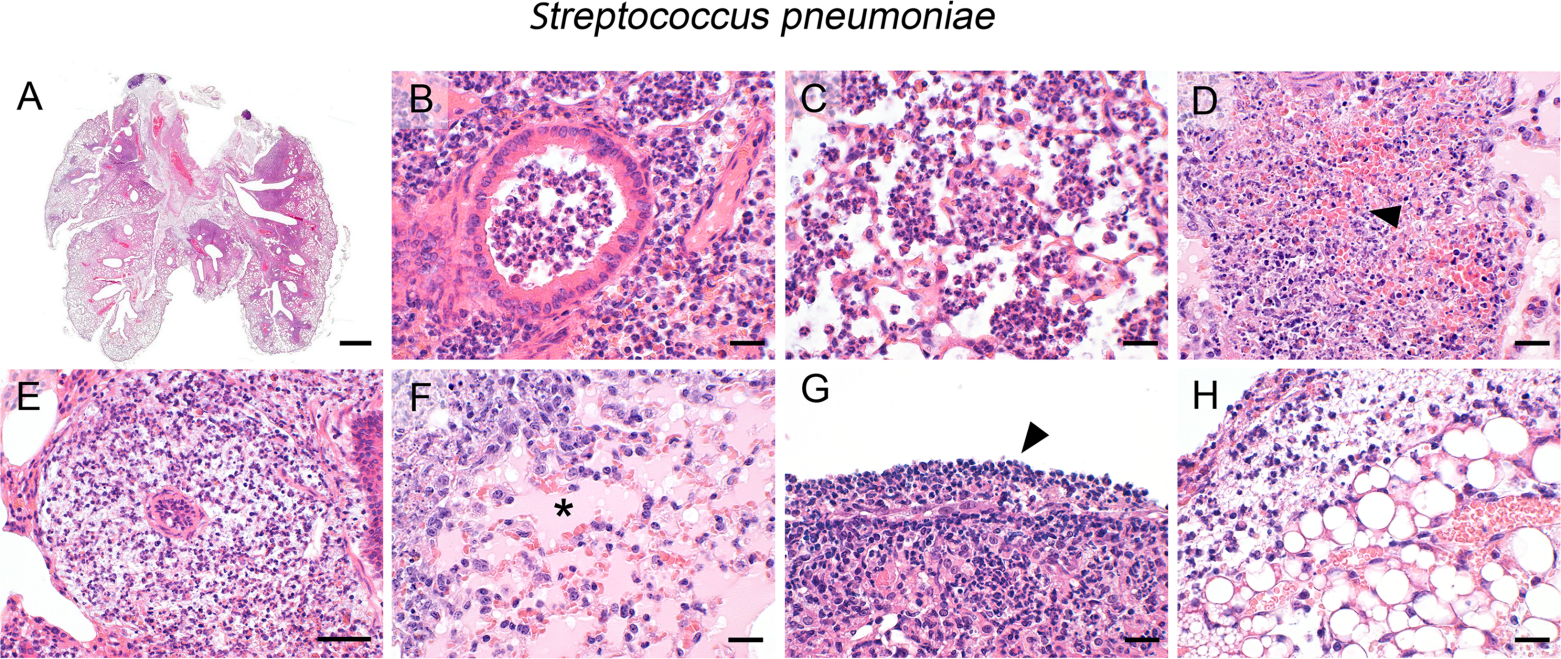
Infection with *S*. *pneumoniae*. (A—H) Lung histology of mice after transnasal infection with *S*. *pneumoniae* (5 x 10^6^ CFU/ mouse) revealed widely expansive (A), suppurative to necrotizing bronchopneumonia (a-d), predominantly infiltrated by neutrophils within bronchial lumina (B) and alveoli (C) and large areas of necrosis and hemorrhage (D, arrowhead). Additional features included marked neutrophilic infiltration and edema of perivascular spaces (E), severe alveolar edema (F, asterisk) and necropurulent pleuritis (G, arrowhead) and steatitis (H). (A—H) Representative images are shown. Bars (A), 1 mm; (B—D, F—H), 20 μm; (E), 50 μm.

Transnasal infection of mice with *S*. *pneumoniae*, serotype 3 resulted in a broad spectrum of tissue lesions and immune cell infiltrations that are typical of aerogenic bacterial pneumonia. Specific for this model, lesions widely expanded down to the periphery of the lung lobes (Fig 1A) with inflammation closely surrounding the airways and blood vessels. Pneumococcal spread led to an early immune response which was mainly characterized by predominantly intrabronchial (Fig 1B) and intraalveolar (Fig 1C) infiltrations of neutrophils provoking a lobular, suppurative bronchopneumonia with consolidation of affected lung areas. Large areas of coagulation and liquefaction necrosis (Fig 1D, arrowhead) as indicated by cellular fragmentation, decay, and loss of cellular details, accumulation of cellular and karyorrhectic debris as well as karyorrhexis, karyopyknosis, and karyolysis with consecutive hemorrhage were also present. The perivascular interstitium was widely expanded by edema due to vascular leakage [53] with massive extravasation of neutrophils recruited into perivascular spaces (Fig 1E). Furthermore, suppurative and necrotizing vasculitis accompanied by hyaline thrombi within small-sized blood vessels were occasionally present, indicating early histological evidence of incipient septicemia. Increased pulmonary vascular permeability [53] also led to expanded areas of protein-rich alveolar edema which presented as homogenous, lightly pink material within the alveolar spaces in the HE stain (Fig 1F, asterisk). A distinctive histopathological feature of pneumococcal pneumonia was the occurrence of massive suppurative to necrotizing pleuritis (Fig 1G, arrowhead) and steatitis (Fig 1H) with widespread dispersion of bacteria into the thoracic cavity, likely accounting for the painful and morbid clinical behavior with rapid progression in affected mice. Myriads of pneumococci were clearly visible as bluish to purple dots of approximately 1 μm in size in the standard HE stain, mostly located on the pleural surface, in the mediastinal adipose tissue or within perivascular spaces in the lungs.

### S. aureus

**Fig 2:**
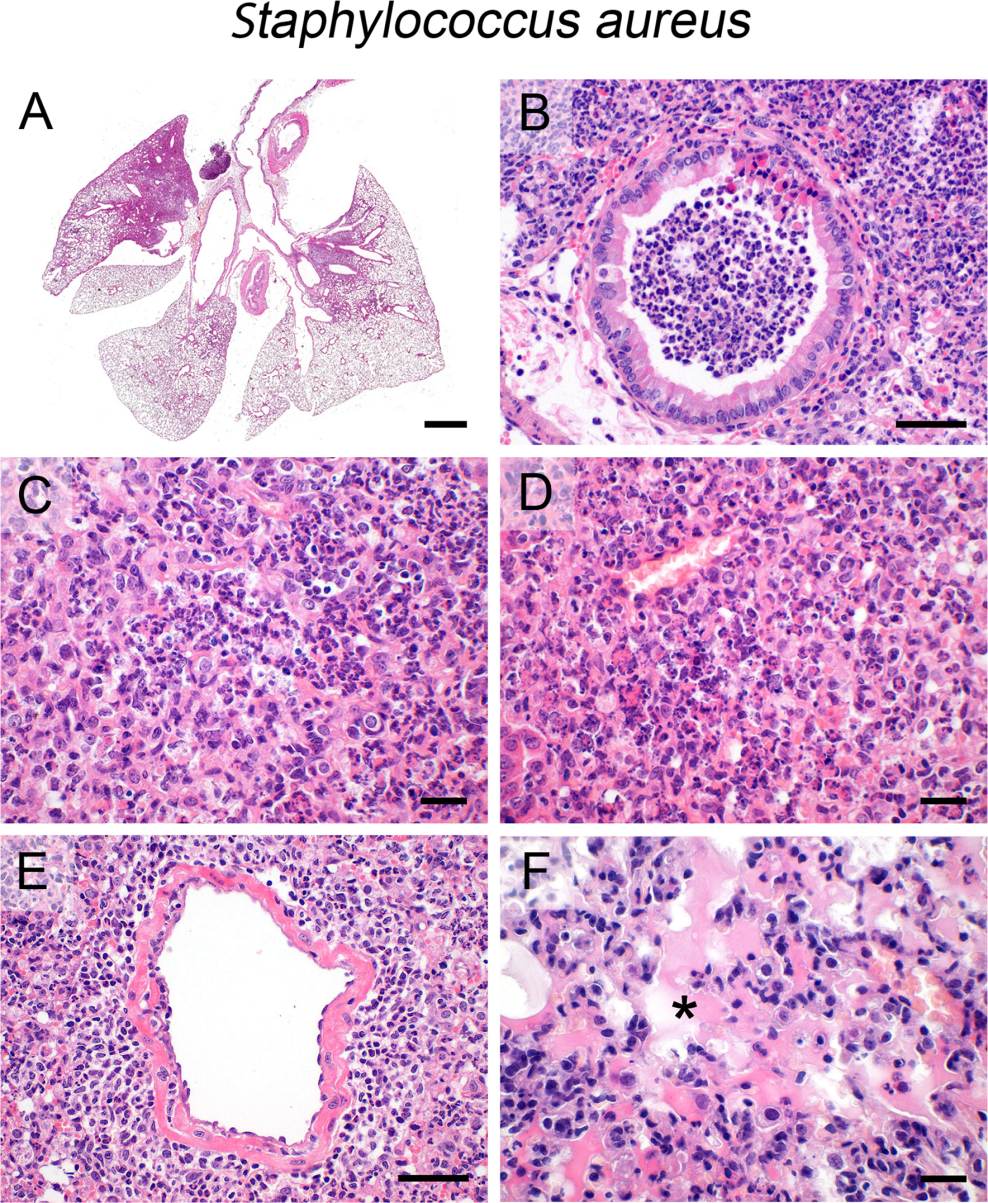
Infection with *S*. *aureus*. (A—F) Transnasal infection of mice with *S*. *aureus* (5 x 10^7^ CFU/ mouse) resulted in non-expansive (A), suppurative (B, C) to necrotizing (D) bronchopneumonia with infiltration of neutrophils and lymphocytes into perivascular spaces (E) and protein-rich alveolar edema (F, asterisk). (A–F) Representative images are shown. Bars (A), 1 mm; (C, D, F), 20 μm; (B, E), 50 μm.

In contrast, transnasal infection with *S*. *aureus* resulted in multifocally extensive but non-expansive bronchopneumonia predominantly located near the lung hilus (Fig 2A), affecting the bronchi, alveoli and interstitium. The main inflammatory cell population consisted of neutrophils, leading to mainly suppurative (Fig 2B and 2C) lesions with a tendency towards abscess formation. In contrast to pneumococci, macrophages were also present albeit at lower numbers than neutrophils. (Fig 2C). Similar to *Klebsiella* and streptococci, large areas of necrosis and hemorrhage (Fig 2D) were present. The perivascular areas were predominantly infiltrated by lymphocytes and fewer neutrophils (Fig 2E). Compared to the *S*. *pneumoniae* model mentioned above [53], vascular permeability seemed only slightly increased as reported before [38] and perivascular edema (Fig 2E) as well as protein-rich alveolar edema (Fig 2F, asterisk) were also present albeit to a lesser extent. Neither pleuritis nor steatitis were observed consistent with a rather favorable clinical outcome under the conditions used. Furthermore, staphylococci were largely undetectable by HE stain which was possibly due to the low bacterial spread within the lungs.

### K. pneumoniae

**Fig 3:**
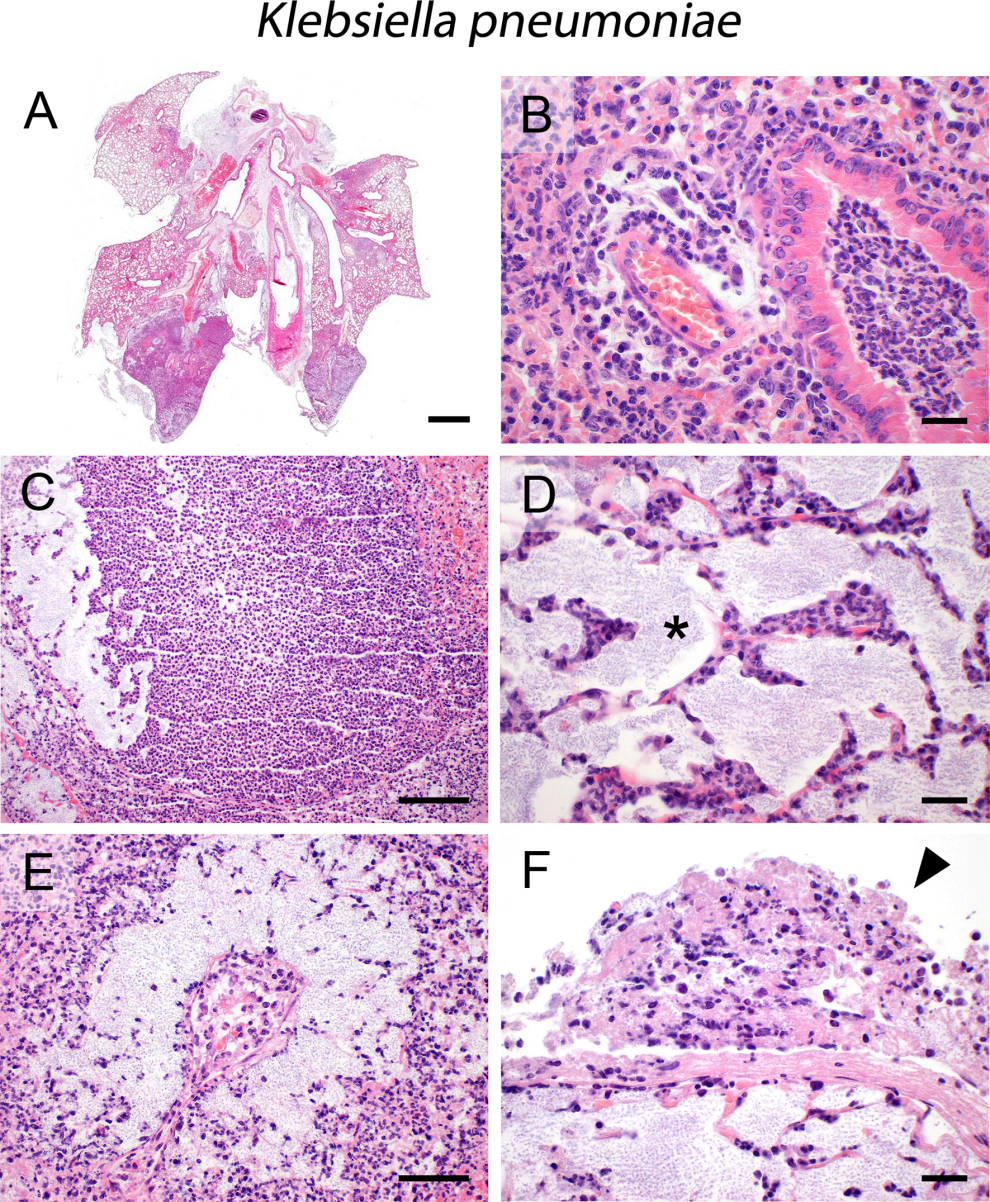
Infection with *K*. *pneumoniae*. (A—F) Intratracheal infection of mice with *K*. *pneumoniae* (3.5 x 10^5^ CFU/ mouse) resulted in expansive (A), suppurative (B) to abscessing (C) bronchopneumonia as well as neutrophilic interstitial pneumonia (D) with severe alveolar (D, asterisk) and perivascular (E) edema, massive fibrinopurulent and necrotizing pleuritis (F, arrowhead) and steatitis. (A–F) Representative images are shown. Bars (A), 1 mm; (B, D, F), 20 μm; (E), 50 μm; (C), 100 μm.

Intratracheal infection of mice with *K*. *pneumoniae* resulted in severe widely expansive bronchopneumonia with increased lesion severity in the lung periphery (Fig 3A). Recruited immune cells predominantly consisted of neutrophils, leading to suppurative (Fig 3B) to abscessing (Fig 3C) bronchopneumonia with hemorrhage and necrosis as well as neutrophilic interstitial pneumonia (Fig 3D) in less affected areas. Increased vascular permeability as reported [40] was associated with massive alveolar (Fig 3D, asterisk) and perivascular edema (Fig 3E), admixed with myriads of bacteria easily recognizable as purple dots in the HE stain. Suppurative to necrotizing vasculitis, pleuritis (Fig 3F, arrowhead), and steatitis were also present and associated with marked bacterial spread and the rapid lethal clinical outcome.

### A. baumannii

**Fig 4:**
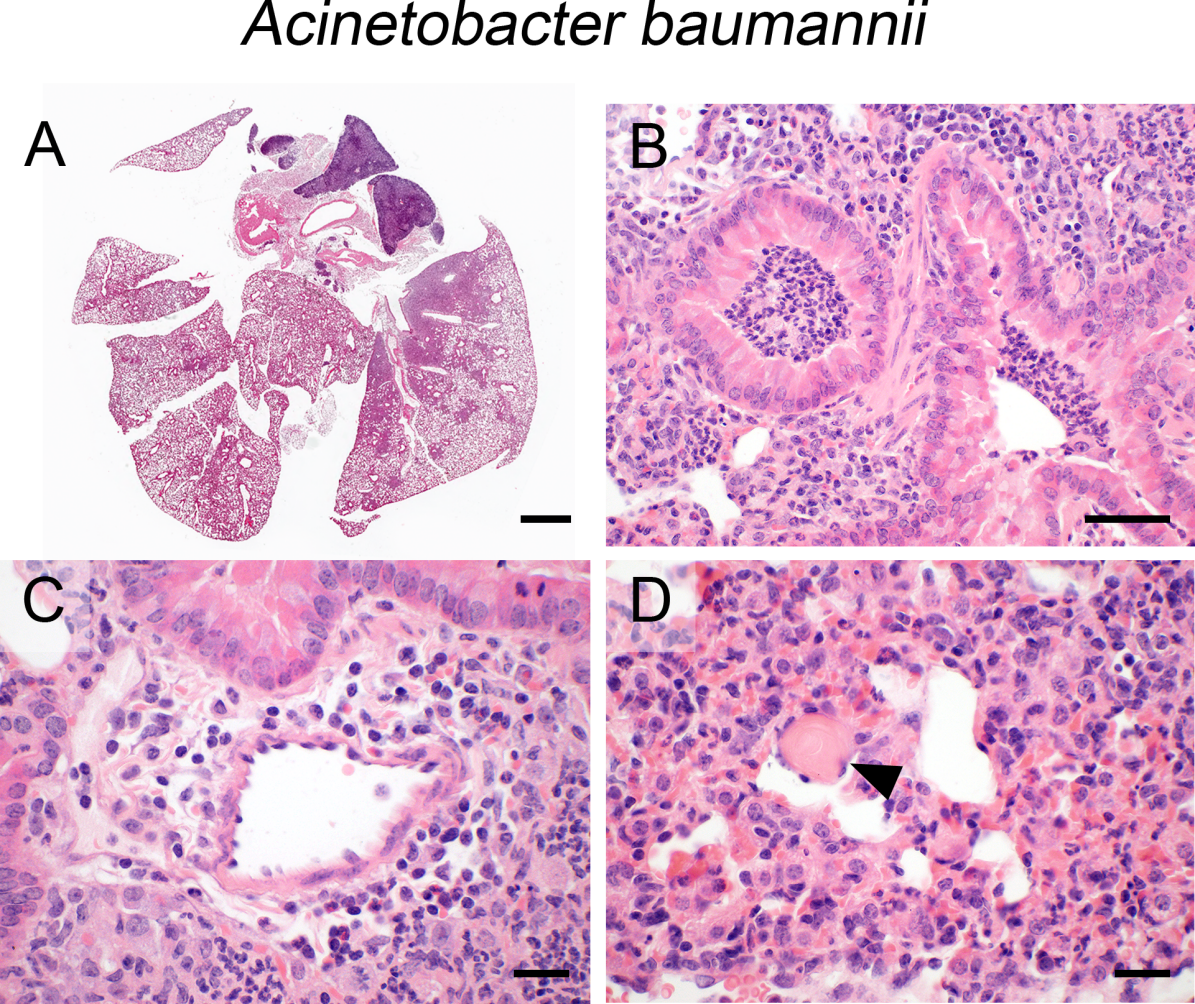
Infection with *A*. *baumannii*. (A—D) Lung histology of mice after transnasal infection with *A*. *baumannii* (5 x 10^8^ CFU/ mouse) revealed expansive (A), suppurative (B) to abscessing bronchopneumonia with perivascular inflammation and edema (C) and vascular thrombosis (D, arrowhead). (A—D) Representative images are shown. Bar (A), 1 mm; (C, D), 20 μm; (B), 50 μm.

After transnasal infection with *A*. *baumannii*, mice developed a widely expansive (Fig 4A) bronchopneumonia with predominantly infiltrating neutrophils causing a suppurative (Fig 4B) to abscessing inflammation with areas of hemorrhage within alveoli and interstitium and large areas of parenchymal necrosis as well as alveolar edema. Perivascular spaces had mild to moderate edema and infiltration of lymphocytes and neutrophils (Fig 4C). Vascular thrombosis was a common change (Fig 4D, arrowhead) in small-sized blood vessels. Similar to staphylococci, *Acinetobacter* was invisible by HE stain and neither pleuritis nor steatitis were present.

### L. pneumophila

**Fig 5:**
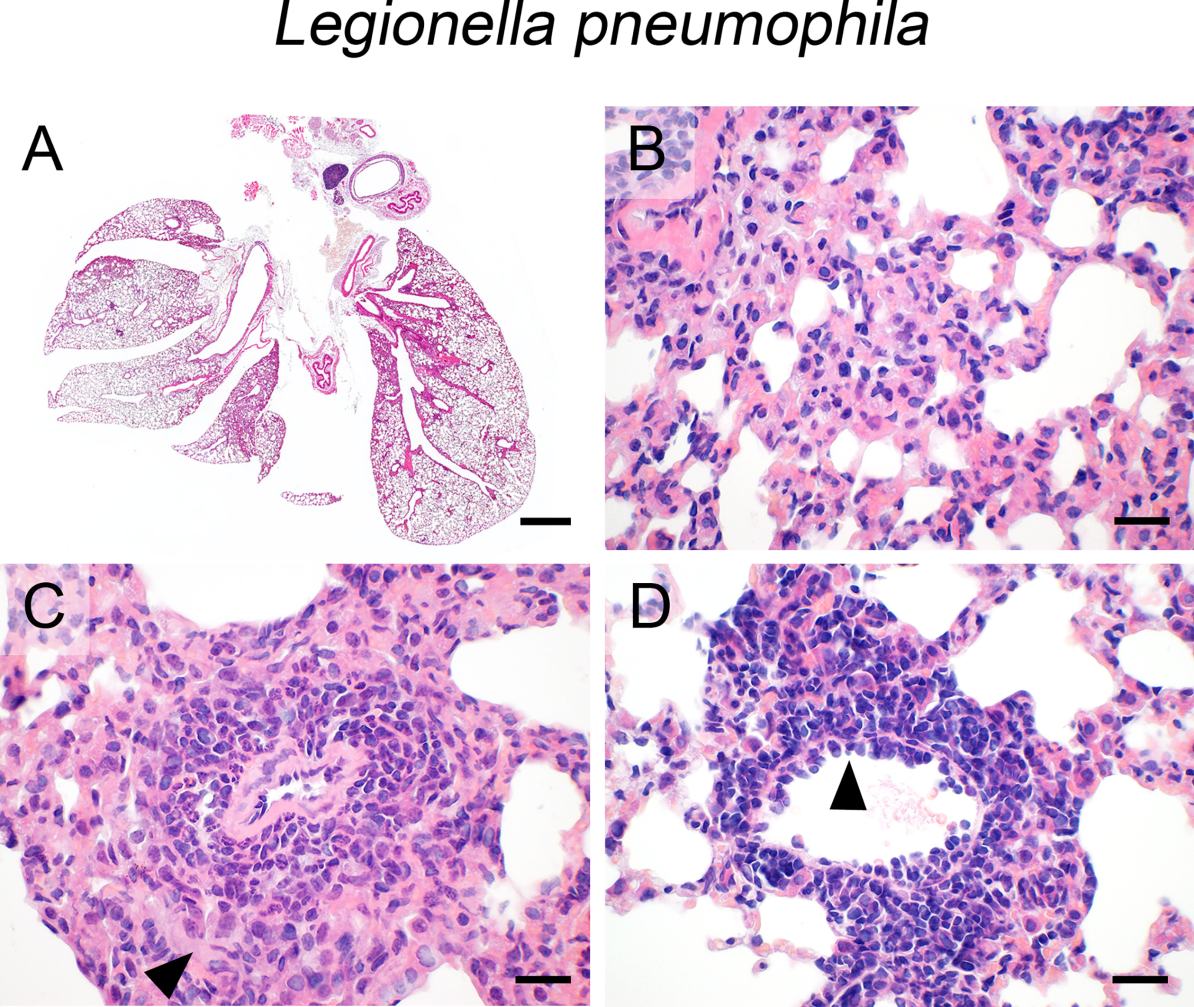
Infection with *L*. *pneumophila*. (A—D) At 48 hours and 6 days post transnasal infection with *L*. *pneumophila* (1 x 10^6^ CFU/mouse), mice developed non-expansive (A), necrotizing to histiocytic interstitial pneumonia (B) with granuloma formation around blood vessels (C, arrowhead) and prominent lymphocytic perivascular cuffing with highly activated endothelial cells (D, arrowhead) at the 6 day-time point. (A—D) Representative images are shown. Bar (A), 1 mm; (B—D), 20 μm.

Transnasal infection of mice with *L*. *pneumophila* resulted in slightly different lesion patterns depending on the time point of examination after infection. At 48 hours after infection, non-expansive interstitial pneumonia was found in close proximity to the hilus (Fig 5A) with prominent alveolar wall necrosis (Fig 5B). At the 6 day-time point, specifically the numbers of infiltrating macrophages were clearly increased, leading to accentuated perivascular granuloma formation (Fig 5C, arrowhead). Here, marked lymphocytic cuffing of most blood vessels as well as highly activated endothelial cells (Fig 5D, arrowhead) were observed. At both time points, neither pleuritis nor steatitis were present. Bacteria were invisible in the HE stained sections.

### E. coli

**Fig 6:**
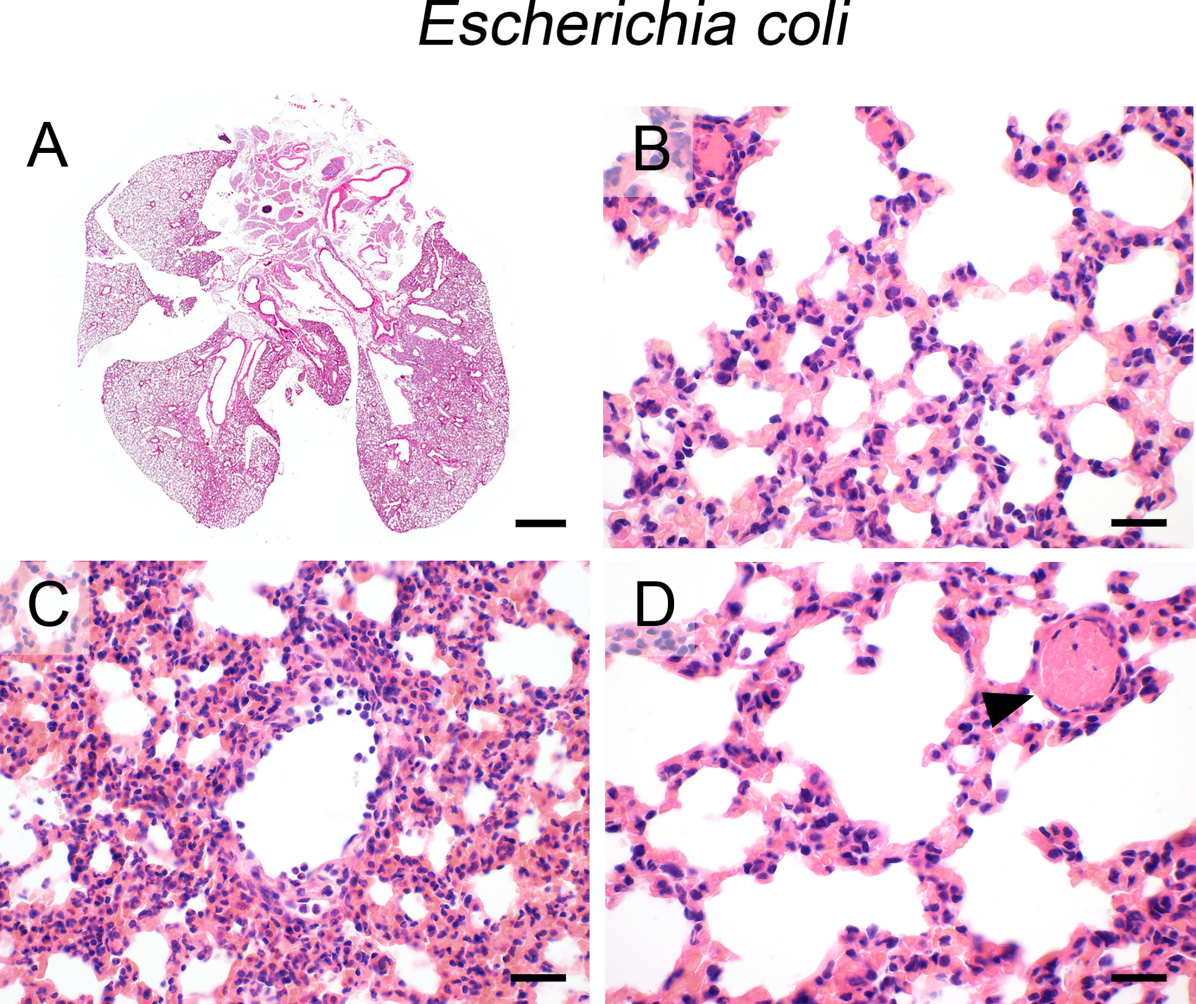
Infection with *E*. *coli*. (A—D) Following intraperitoneal infection with *E*. *coli* (5 x 10^8^ CFU/ mouse), mice developed diffuse (A), neutrophilic interstitial (B) and perivascularly accentuated (C) pneumonia with marked vascular thrombosis (D, arrowhead). (A—D) Representative images are shown. Bar (A), 1 mm; (B—D), 20 μm.

After intraperitoneal infection, the hematogeneous spread of *E*. *coli* to the lungs had resulted in diffuse, interstitial suppurative pneumonia, diffusely affecting the entire lungs, modelling sepsis-induced ALI (Fig 6A). The interalveolar interstitium was heavily infiltrated with neutrophils (Fig 6B) with most prominent aggregation around blood vessels (Fig 6C), consistent with bacterial entry via the circulation. Numerous hyaline thrombi were present within small-sized blood vessels (Fig 6D, arrowhead), suggestive of disseminated intravascular coagulopathy (DIC) due to bacterial septicemia. Large, rod-shaped bacteria were easily detectable only outside the lungs, mostly present in the adipose tissue surrounding the esophagus, possibly due to local spread of *E*. *coli* via the abdominal cavity.

### MERS-coronavirus

**Fig 7:**
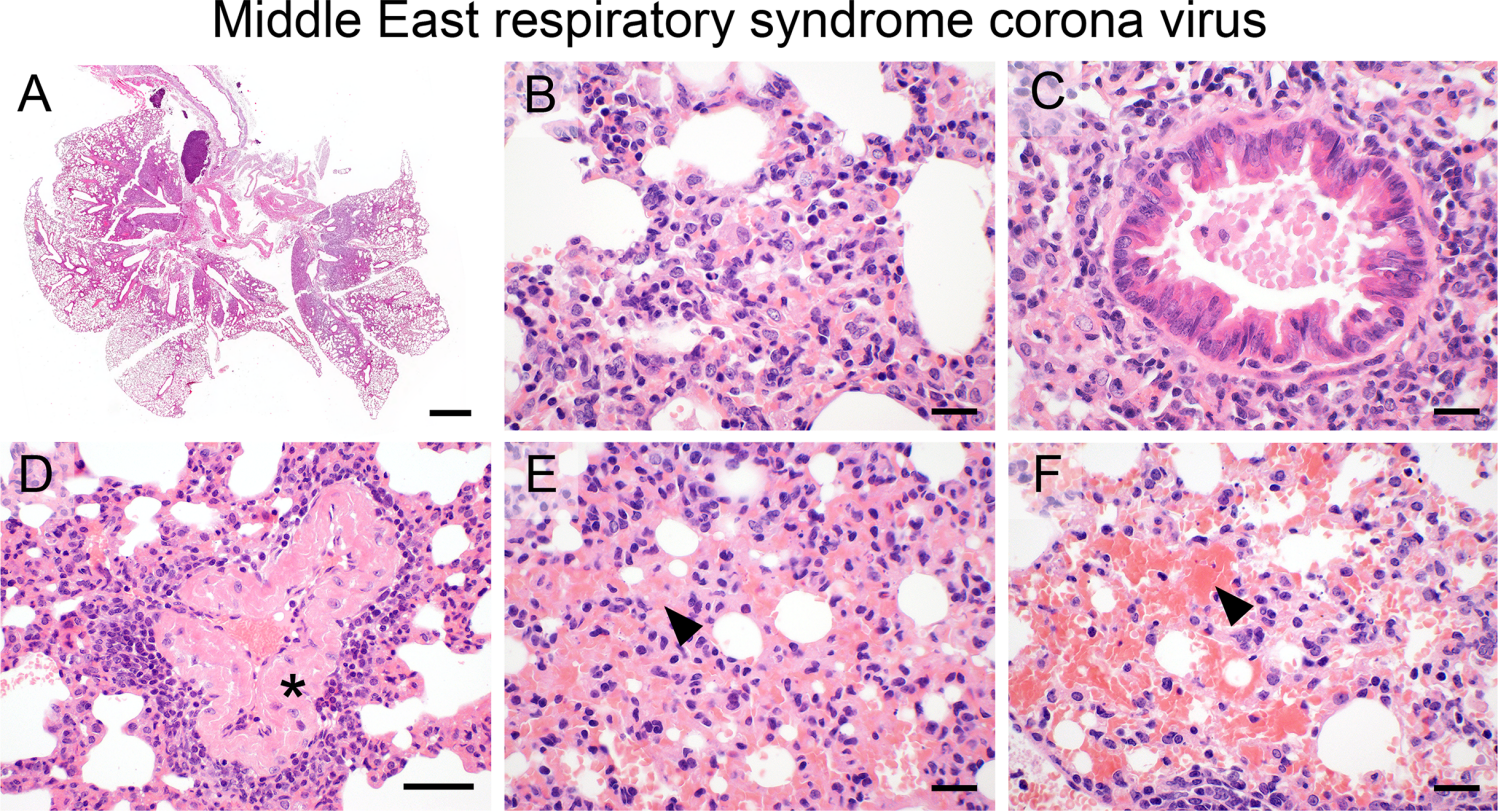
MERS-coronavirus. (A—F) Transnasal infection with MERS-CoV (7 x 10^4^ TCID_50_/ mouse) resulted in expansive (A), necrotizing, mixed-cellular bronchointerstitial pneumonia (B) with peribronchial and perivascular infiltration of lymphocytes (C, D), marked fibrinoid degeneration of blood vessels (D, asterisk), alveolar edema (E, arrowhead) and hemorrhage (F, arrowhead). (A–F) Representative images are shown. Bar (A), 1 mm; (B, C, E, F), 20 μm; (D), 50 μm.

Transnasal infection with MERS-CoV following adenoviral transduction of human DPP4 yielded an expansive, (Fig 7A) interstitial pneumonia with severe alveolar epithelial cell necrosis and infiltration of mainly macrophages, lymphocytes, and fewer neutrophils (Fig 7B). Only moderate peribronchial (Fig 7C) and perivascular (Fig 7D) lymphocytic infiltrations were present while most venous blood vessels had marked fibrinoid degeneration and necrosis of vascular walls (Fig 7D, asterisk). Additional hallmarks of MERS-CoV infection were large areas of protein-rich alveolar edema (Fig 7E, arrowhead), pronounced hemorrhage within perivascular and alveolar spaces, and interstitium (Fig 7F, arrowhead), and the formation of hyaline thrombi within small-sized blood vessels.

### Influenza A virus

**Fig 8:**
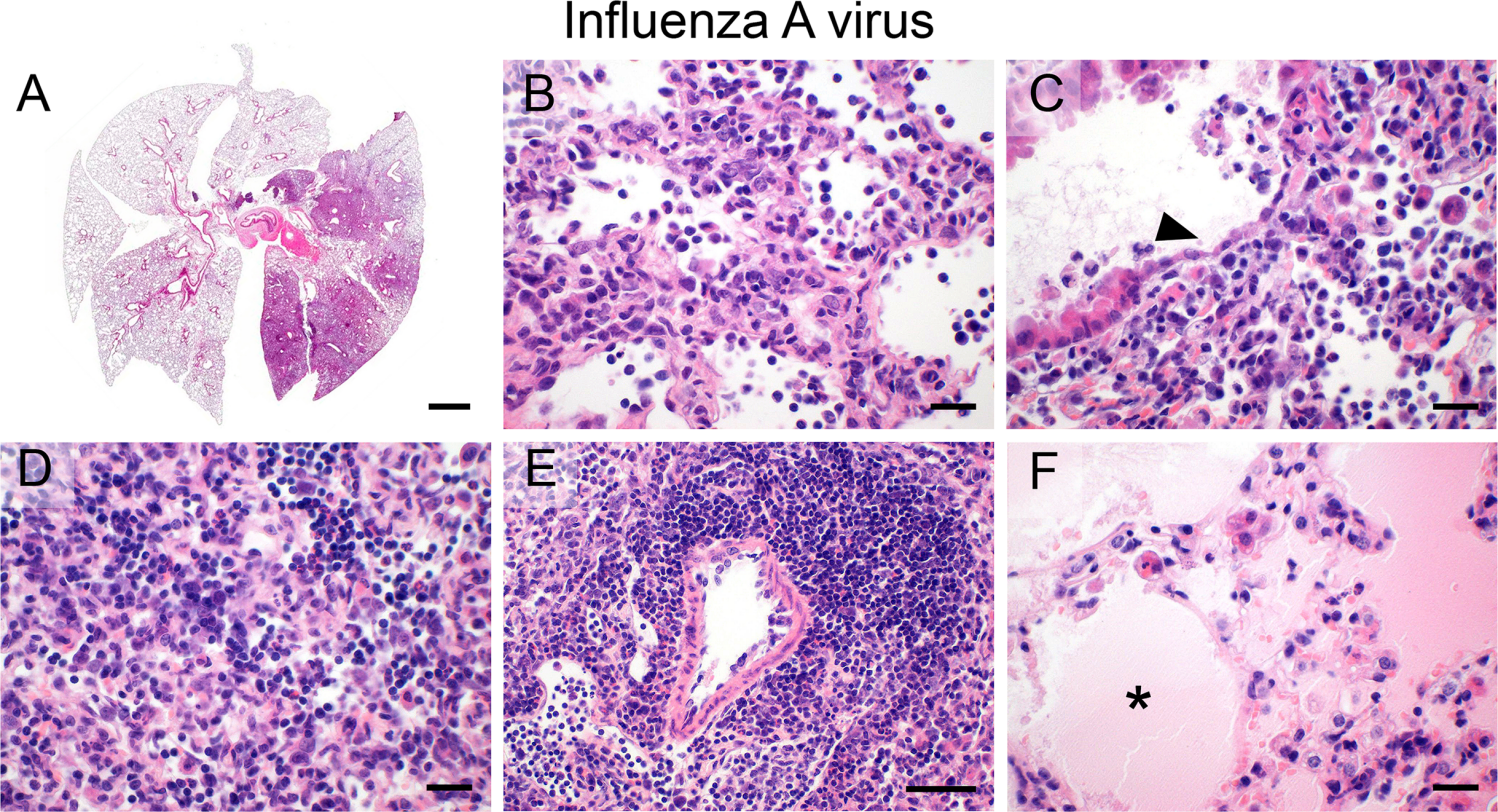
Influenza A virus. (A–F) After transnasal infection, IAV (100 PFU/ mouse) resulted in diffuse (A), necrotizing (B), bronchointerstitial pneumonia (B, C) with marked necrosis and sloughing of bronchial epithelial cells (C, arrowhead), lympho-histiocytic intraalveolar and interalveolar interstitial infiltration (D), prominent perivascular cuffing of lymphocytes (E) and protein-rich alveolar edema (F, asterisk). (A–F) Representative images are shown. Bar (A), 1 mm; (B—D, F), 20 μm; (E), 50 μm.

After transnasal infection with IAV, mouse lungs displayed a diffusely distributed bronchointerstitial pneumonia restricted to single lung lobes only (Fig 8A). Alveolar necrosis was prominent and alveolar septae were diffusely distended by infiltrating inflammatory cells (Fig 8B). Bronchial epithelial cells were markedly necrotic (Fig 8C, arrowhead) and extensively scaled off into the bronchial lumen. Alveoli and interstitium were filled with macrophages and lymphocytes as major effector cells (Fig 8D) and prominent perivascular lymphocytic cuffing (Fig 8E) was a characteristic change. Furthermore, large areas of alveolar edema (Fig 8F, asterisk) and, albeit to a much lesser extent, areas of hemorrhage within alveoli and interstitium were present, suggesting vascular damage and increased permeability.

### Superinfection of influenza A virus pneumonia with *S*. *pneumoniae*

**Fig 9:**
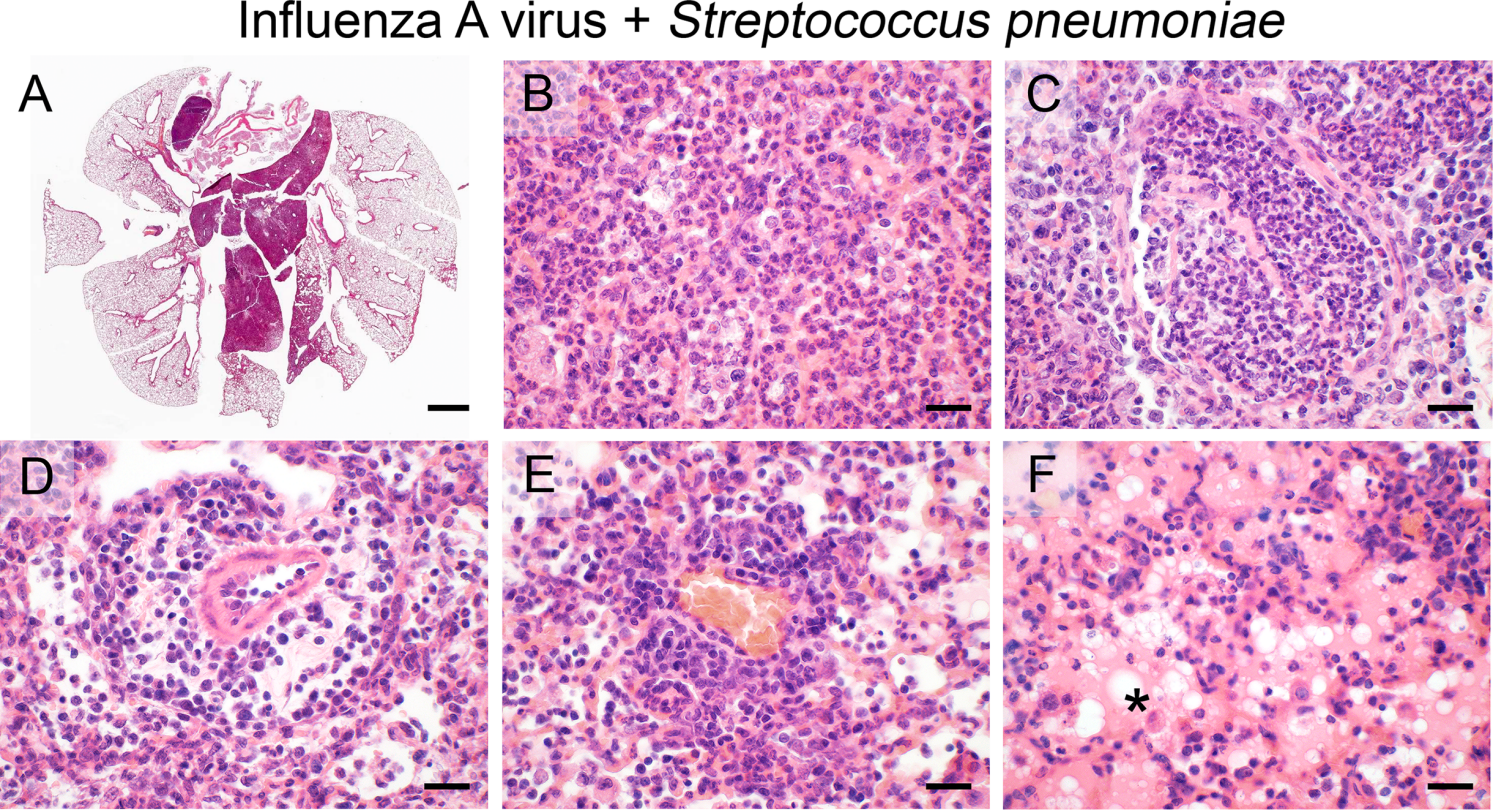
Superinfection of influenza A virus pneumonia and *S*. *pneumoniae*. (A–F) Superinfection with *S*. *pneumoniae* (1 x 10^3^ CFU/ mouse) of mice that had been infected with IAV (40 PFU/ mouse) earlier resulted in severe expansive (A), suppurative bronchopneumonia (B) with marked necrosis of bronchi which were filled up with pus (C). In addition, perivascular infiltration of neutrophils, lymphocytes and monocytes with prominent edema (D) as well as mild purely lymphocytic, dense perivascular cuffing in other areas (E) and protein-rich alveolar edema (F, asterisk) were present. (A–F) Representative images are shown. Bars (A), 1 mm; (B—F), 20 μm.

When mice had been infected with IAV prior to infection with *S*. *pneumoniae*, a combination and exponentiated phenotype of both models was observed 24 hours later. Lesions were widely expansive to the lung periphery but restricted to single lung lobes pre-damaged by IAV (Fig 9A). The character of pneumonia included massive infiltration of neutrophils into alveoli (Fig 9B) and bronchi, typical features of severe, suppurative bronchopneumonia. Bronchial epithelium was almost entirely necrotic and bronchi were filled up with pus (Fig 9C). Perivascular spaces were edematous and infiltrated by neutrophils and lymphocytes (Fig 9D) whereas only mild lymphocytic perivascular cuffing (Fig 9E) was present. A severe protein-rich alveolar edema was seen, similar to that seen in the *S*. *pneumoniae* mono-infection (Fig 9F). Pneumococci were difficult to visualize by HE stain, possibly due to the lower infectious dose used here when compared to the mono-infection.

### Distributions, expansions and symmetry

**Fig 10:**
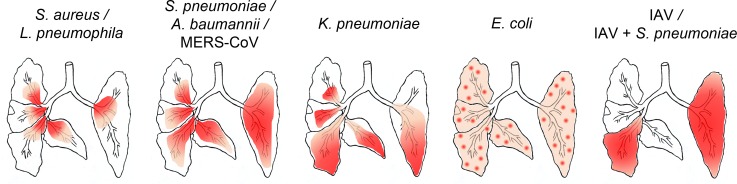
Distributions, expansions and symmetries of affected lung parenchyma. Infection of murine lungs with different bacterial or viral pathogens resulted in striking differences in the distributions, expansions and symmetries of pneumonic lesions that were highly reproducible for each pathogen. Mainly centrally located lesions induced by *S*. *aureus* and *L*. *pneumophila* remained close to the hilus with no trend towards peripheral expansion. Infection with *S*. *pneumoniae*, *A*. *baumannii* and MERS-CoV resulted in lesions closely surrounding the airways and blood vessels close to the central major airway segments with centrifugal expansion towards the periphery. In contrast, lesions induced by *K*. *pneumoniae* were mostly located in the periphery of the lobes and airways and much weaker adjacent to the hilus. Hematogenous infection with *E*. *coli* was associated with entirely diffuse distribution of lesions affecting the whole lungs with myriads of inflammatory hot spots, commonly surrounding blood vessels. IAV-induced lesions were restricted to individual lung lobes with a rather homogeneous distribution within affected lobes. Which lobes were affected followed a rather random and inconsistent pattern. Superinfection of *S*. *pneumoniae* into an IAV-pneumonia resulted in a pattern virtually identical to that seen after IAV infection alone. Except for *E*. *coli* induced pneumonia, virtually all lung lesions were distributed asymmetrically between the left and right lung halves with no tendency of either half to be more often or more strongly affected.

Prior to processing for histopathology, small tissue samples from experimentally infected mouse lungs are commonly removed for molecular analyses of gene and/ or protein expression or other readout systems to receive additional information. To obtain representative data from such samples that can be correlated with the histological changes, it is crucial to know about the homogeneity of the distribution of lesions. Also, some experimental protocols recommend to use the left and right halves of the lungs, respectively, for different analytical procedures, again anticipating lesion homogeneity and symmetry. However, when we analyzed the distributions and bilateral symmetry of lung lesions for each of the infection models, we found a wide spectrum of distinct distributions and asymmetries (Fig 10). In principle, lesion distributions followed the route of pathogen entry into the lungs. However, the tendencies to spread towards the periphery of the lobes after aerogenous infection varied between different pathogens despite similar aerogenous infection routes. Mostly centrally focused lesions induced by *S*. *aureus* and *L*. *pneumophila* remained close to the hilus with no trend towards peripheral expansion. Infection with *S*. *pneumoniae*, *A*. *baumannii* and MERS-CoV resulted in lesions closely surrounding major airway segments with centrifugal expansion towards the periphery. In contrast, lesions induced by *K*. *pneumoniae* were mostly located in the periphery of the lobes and airways and much weaker adjacent to the hilus despite aerogenous infection. Hematogenously-induced sepsis with *E*. *coli* was associated with an entirely diffuse distribution of lesions affecting the whole lung with myriads of inflammatory hot spots, commonly surrounding blood vessels. IAV-induced lesions were restricted to individual lung lobes only with a rather homogeneous distribution within affected lobes. Superinfection of *S*. *pneumoniae* into an IAV-pneumonia resulted in a pattern virtually identical to that seen after IAV infection alone. Except for blood borne *E*. *coli* pneumonia which was consistently and evenly distributed throughout the entire lungs, affected areas in all other models tested here were randomly distributed more or less asymmetrically between the right and left halves of the lungs and also between adjacent lobes (Fig 10).

### Visualization of pathogens using special stains and immunohistochemistry

**Fig 11:**
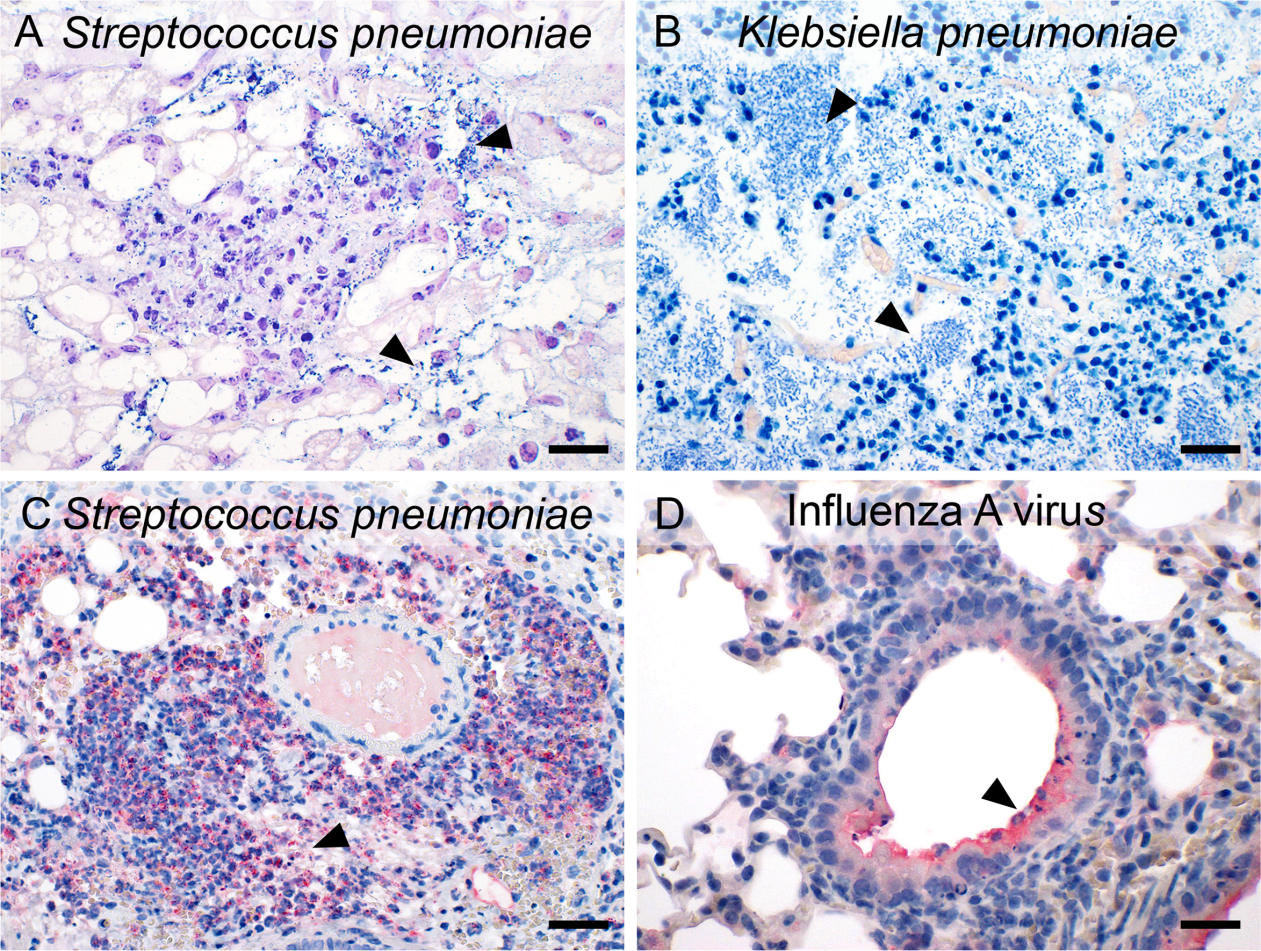
Visualization of pathogens. Pathogens were visualized using special stains commonly used for bacterial detection, including the Gram stain for Gram-positive pneumococci (A, dark blue, arrowhead), and Giemsa stain of *Klebsiella* (B, light blue, arrowhead). Immunohistochemical staining methods were employed to localize specific antigens of *S*. *pneumoniae* (C, red, arrowhead) and IAV (D, red, arrowhead). Triamino-tritolyl-methanechloride (Neufuchsin) was used as chromogen (red) and hematoxylin (*blue) as* counterstain. Bars (a–d), 20 μm.

For more than 100 years, a wide range of special stains have been used for the histological visualization of pathogens and other relevant structures, based on their more or less specific affinities to certain dyes. Here, Gram stain modified by Brown and Brenn was used for the visualization of Gram-positive bacteria, including *S*. *pneumoniae* (Fig 11A, arrowhead), as easily recognizable, dark blue cocci. In contrast, Giemsa stain was conducted predominantly for the detection of Gram-negative bacteria such as *K*. *pneumoniae* (Fig 11B, arrowhead) which then turned into light blue to greenish rods.

For more specific pathogen detection on slides, particularly for viruses, immunohistochemistry is the method of choice. Here, *S*. *pneumoniae* and IAV were detected by immunohistochemistry using specific anti-*S*. *pneumoniae* or anti-IAV antibodies, respectively. *S*. *pneumoniae*-positive signals were obtained as myriads of red cocci predominantly in the perivascular interstitium (Fig 11C), within neutrophils in alveoli and interstitium, and on pleural surfaces as well as in mediastinal adipose tissue. In addition, pneumococci were also visualized in the marginal sinuses of tracheal lymph nodes, both in macrophages and extracellularly. IAV antigen was localized to the apical surface and cytosol of intact and necrotic bronchial epithelial cells (Fig 11D) and within alveolar macrophages.

### Selection of criteria suitable for scoring systems for each pathogen

The diversity of lesions and in particular the presence or absence of specific patterns in several of the models used (Table 1) strongly suggested that a uniform scoring scheme for the semiquantification of mouse pneumonia is inconceivable. Instead, scoring systems should take into account the more or less pathogen-specific lesion patterns that can be distilled from the comparative characterizations given above. For this purpose, we carved out the most characteristic lesion patterns that appear suitable for the development of specific scoring schemes for each model (Table 2).

## Discussion

Different mouse models of acute pneumonia differ widely, with an obvious strong dependence on pathogen-specific features of virulence and spread, route of infection, infectious dose and other factors. Here, we provide a detailed descriptive overview of histopathological features and distributions of lesions within infected lungs and compare them between nine relevant and commonly used infection models at their peaks of injury and inflammation. The models employed here all represent well established protocols that have been optimized and successfully used in previous studies, with model-specific variations in infection doses, routes of pathogen administration and analyzed time points [37–42, 51, 52, 54–56]. Our model-specific description parameters (Table 2) provide a rational for the selection of histopathological quantification criteria, in order to best reflect the model-specific lesion and distribution characteristics, which appear to be most relevant. Clearly, the severity of lesions in terms of outcome of quantification systems will depend on several other factors that will have to be addressed separately in each model, such as the infection dose, time point of examination or therapeutic interventions.

The model-associated characteristics of tissue lesions and immune cell infiltrates are widely consistent with well-established properties of the different pathogens used. For example, the destructive tissue damage with mostly neutrophilic infiltrations as seen in *S*. *pneumoniae*, *S*. *aureus*, and *A*. *baumannii*, are typically seen with extracellular bacteria that express cytotoxic virulence factors, such as pneumolysin and hydrogen peroxide [61] from *S*. *pneumoniae* or immunogenic cell wall components such as lipoteichoic acid (LTA) from *S*. *aureus* [62, 63]. On the other hand, the intracellular pathogen *L*. *pneumophila* which primarily infects macrophages [64] resulted in a histiocytic infiltrate at 48 h that developed into granulomatous inflammation at 6 days after infection, typical of a T_H_1-response [65, 66]. However, several of the pathogens used were associated with additional distinctive features. For example, histology revealed marked pleuritis and steatitis due to pathogen invasion into adjacent extrapulmonary tissues after infections with *S*. *pneumoniae* and *K*. *pneumoniae*. This massive bacterial spreading throughout the thoracic cavity was exclusively present in these two models and most likely associated with sepsis, responsible for the rapid clinical progression and unfavourable outcome [41, 67]. However, only *K*. *pneumoniae* had the tendency of abscess formation which was not seen in pneumococcal pneumonia. Infection with *S*. *aureus* and *A*. *baumannii* also resulted in similar lesion patterns, except for *Acinetobacter*-induced lesions widely expanding to the lung periphery while *Staphyloccocus*-induced pneumonia was restricted to the lung hilus. A second difference between the two was the presence of prominent vascular thrombosis in *A*. *baumannii*-induced pneumonia which was absent from S*taphylococcus* pneumonia. The clinical outcome of mice infected with *A*. *baumannii* and *S*. *aureus* was more favourable when compared to infection with *S*. *pneumoniae* or *K*. *pneumoniae* [38] which may be explained by the lack of bacterial spreading throughout the thoracic cavity and adjacent tissues, and possibly sepsis. *E*. *coli* infection was included here as a model for sepsis-associated ALI [29, 30, 68] and consequently induced wide spread vascular thrombosis and vasculitis, most likely due to its blood borne entry into the lungs and concurrent septicemia with disseminated intravascular coagulopathy and associated vascular lesions. Vascular thrombosis with or without vasculitis was also observed in other models, including *S*. *pneumoniae*, *A*. *baumannii* and MERS-CoV, however, to a much lesser extent and only within the most strongly affected areas. MERS-CoV and IAV-associated lesions clearly reflected the known cellular tropisms of these viruses with necrosis of alveolar walls or bronchial epithelial cells, respectively, being the most characteristic histopathologic features [69–72]. Typical of virally induced lesions, the inflammatory cell infiltrates in MERS-CoV and IAV infections were dominated by lymphocytes with no or only few neutrophils. Nevertheless, the two viral models could be clearly distinguished from each other by additional histological characteristics. Only the MERS-CoV infection led to a marked vascular phenotype with necrosis and degeneration of blood vessels, vasculitis, and consecutive vascular thrombosis as well as pronounced hemorrhages [69, 73]. In contrast, IAV-induced pneumonia did not display any of these features, but was dominated by marked perivascular lymphocytic cuffing and alveolar edema [42, 74]. Subsequent superinfection with low-dose *S*. *pneumoniae* potentiated the severity of the IAV-induced lesions and aggravated the course of pneumonia. However, it did not alter the principal histological characteristics of IAV-pneumonia. The patterns seen after single infection with *S*. *pneumonia* were not repeated in this superinfection model, likely owing to the much lower inoculation dose which is usually rapidly cleared from virus-naive lungs.

When the distributions of lesions were compared among the 9 models tested, four distinct patterns could be clearly distinguished. The most common pattern, where lesions were focused around central airways and blood vessels close to the lung hilus with the periphery less or not affected can likely be explained by the aerogenous route of infection and pathogen entry. The opposite pattern characteristic of *K*. *pneumoniae* infection where the periphery of the lobes was more strongly affected than their hilus areas despite a similar aerogenous route of infection may be due to the aerosolic intratracheal application [75] of these bacteria which is typical of and necessary in this model [41, 57–59]. These differences are therefore more likely attributable to the model-specific route of infection rather than pathogen-specific properties. Similarly, the very homogenous distribution of *E*. *coli* induced pneumonia likely followed the diffuse blood borne entry of the pathogen into the lungs after intraperitoneal infection. Again unique among the pathogens tested here, the IAV-associated pattern affected entire but only select lung lobes with almost complete sparing of others. This distribution probably followed a random spread of the virus along major airways but why some lobes remained virtually unaffected after transnasal infection remains hard to explain. Apart from helping to understand differences in pathogen spread, the uneven and often quite asymmetrical distributions have a tremendous impact in practical terms when acute mouse pneumonia is sampled for molecular studies. When quantitative data on mRNA or protein expression levels or other biochemical information are to be compared with one another or with tissue lesions, it is imperative that only identically affected areas are compared. Since this is impossible to predict or recognize on the macroscopical level for most models, the practice of sampling different regions of such lungs for different readout systems appears highly problematic.

Another implication of the distinct lesion characteristics, immune cell reactions and distributions among the different models appears highly relevant for histological scoring systems that aim at first quantitative comparisons [45]. To narrow down the list of parameters appropriate for each pathogen and exclude features that are likely irrelevant for some of the models, we selected 23 single histopathologic criteria for the design of semiquantitative scoring systems suitable for each model. These criteria are partly composed of standard parameters such as the determination of the affected lung area, the distribution of lesions or the type of pneumonia induced. However, numerous other and more model-specific parameters were identified which precisely describe particular aspects and allow for a differentiation between the models, such as the presence of perivascular edema, vascular thrombosis, pleuritis or steatitis. Appropriate scoring systems may thus encompass more general parameters when different pathogens are compared to one another or more pathogen-specific parameters in case specific pathogen features are in the focus of an experiment. For example, some of the parameters selected here have proven helpful in the discovery and semiquantification of different phenotypes of mouse pneumonia following genetic engineering of pathogens or mice [38, 51, 76]. However, scoring systems that claim universality for all mouse models of acute pneumonia seem neither generally applicable nor meaningful for all specific experimental goals. Even the list of 23 parameters selected here may become inappropriate or insufficient when genetic changes on the pathogen or host side may result in different types of lesions, immune cell responses, time courses or other relevant features. In those cases, the list selected here may have to be adjusted or extended to better meet the specific challenges of each new study.

As standard hematoxylin and eosin (HE) staining of tissue sections failed to visualize most pathogens, traditional special stains as well as immunohistochemical techniques were employed, depending on specific staining properties of the pathogens and the availability of appropriate antibodies. While *S*. *pneumoniae*, *K*. *pneumoniae* and *E*. *coli* were easily visible in HE stained tissue sections in areas with low density of inflammatory cells, e.g., in perivascular spaces, they were very difficult to identify in heavily infiltrated and consolidated lung parenchyma. In contrast, *S*. *aureus*, *A*. *baumannii*, *L*. *pneumophila* and both viruses were entirely invisible by HE staining and thus had to be visualized by appropriate histotechnical stains or immunohistochemistry. Both approaches will likely also allow for a rough quantification of pathogen numbers in tissues when appropriate image analysis tools are used.

In this first comparative study of its kind, we examined previously established models with their optimized routes of infection, time points, and infection doses and volumes specific for each model to reach peaks of lung injury and inflammation. Variations of such factors can be expected to result in different lesion severities, composition of the cellular infiltrates, and for some models in different expansions of lesions within the lung. Still, the conditions used here are all based on observations that have evolved during extensive previous establishment studies of these models [37–39, 41, 42, 51, 52, 54, 56–58, 60, 77–79]. Among the most important reasons, most human pathogens are not pathogenic for mice under non-experimental conditions and the decisive factor for obtaining a useful pneumonia model appears to be the determination of the appropriate infection dose and route of infection. In addition, the exact time points of tissue analysis after infection had to be determined for virtually all models with care to obtain a useful model, including a precise definition of the strain or variant of the pathogen used [51, 53, 80, 81]. Another variable to consider is the mouse strain used. Except for BALB/c mice which were used in the MERS-CoV infection model here for model-specific reasons [60], all models were conducted with C57BL/6 mice which is among the most commonly used mouse strain in infection research and therefore allows for comparisons with similar studies. However, variations of the strain or genetic background may have a dramatic impact on the type, severity and outcome of inflammation, particularly in innate immune responses [82–84]. Again, the criteria suggested here for scoring procedures should allow to recognize and quantify such differences related to changes in infection dose and volume, time point of examination, strain and age of mice used, pathogen variant and other variables.

Histopathology of the lungs may be complex and requires fundamental knowledge in species-specific anatomy, physiology, organ-specific immunology, pathology, and histotechnical procedures. Furthermore, various background lesions in mice, including strain specific spontaneous degenerative or inflammatory conditions and the possibility of accidental infections unrelated to the experiment should not be confused with experimental outcome. Thus, despite our efforts to specify and simplify the criteria relevant for model-specific assessment and quantification of lesions, it appears crucial that trained histopathology experts be involved in the microscopical examination of mouse lungs [46, 85].

Clearly, in addition to descriptive or semiquantitative histology, a number of other parameters may be useful for quantitative comparisons between experimental groups to determine the role of specific cell types, molecules, and therapeutic interventions, depending on the strategy and goal of the study [44]. Such parameters could include flow cytometric immune cell identifications and quantifications, ELISA or quantitative RT-PCR for the probing of cytokines, chemokines or matrix proteins involved in lung pathology and remodeling, and plaque/colony forming assays for the identification or quantification of pathogens, as previously published for most of the models used here [38, 41, 42, 51–53, 86, 87]. All of these methods, however, lack the spatial resolution that only histological assessments offer. Only the combination of these techniques will lead to a better understanding of the disease in the complex context of the entire lung pathology.

In conclusion, we have identified a spectrum of pathogen- and model-specific lesion characteristics in mouse models of acute pneumonia. Our findings underscore the necessity of model-specific criteria for the accurate histopathological characterization and quantitative assessments of experimental pneumonia. This comparative landscaping of acute mouse pneumonia histology provides a comprehensive framework for future studies on the role of individual pathogen or host factors, complex disease mechanisms, and novel therapeutic strategies that could help to treat pneumonia in human patients.
